# *In vivo* activation of coagulation during human liver transplantation is associated with activation of the intrinsic pathway: an observational cohort study

**DOI:** 10.1016/j.rpth.2025.102872

**Published:** 2025-04-24

**Authors:** Fynn L. Elvers, Jelle Adelmeijer, Sarah Bos, Coen Maas, William Bernal, Ton Lisman

**Affiliations:** 1 Surgical Research Laboratory and Section of Hepatobiliary Surgery and Liver Transplantation, Department of Surgery, University of Groningen, University Medical Center Groningen, Groningen, The Netherlands; 2 Department of Gastroenterology, Treant Hospital, Emmen, The Netherlands; 3 Central Diagnostic Laboratory Research, University Medical Center Utrecht, Utrecht University, Utrecht, The Netherlands; 4 Liver Intensive Therapy Unit, Institute of Liver Studies, King’s College Hospital, Denmark Hill, London, United Kingdom

**Keywords:** blood coagulation, factor XII, hepatectomy, liver transplantation, venous thromboembolism

## Abstract

**Background:**

Patients undergoing hepato-pancreato-biliary surgery experience substantial changes in their hemostatic system. The postoperative risk of venous thromboembolism is high, even in the presence of adequate thromboprophylaxis.

**Objectives:**

As the hemostatic mechanisms underlying the thrombotic risk in these patients are incompletely studied, we aimed to identify the extent of *in vivo* activation of coagulation in relation to the activation of the intrinsic and extrinsic pathways.

**Methods:**

We studied plasma samples before, during, and after surgery from patients undergoing orthotopic liver transplantation (OLT; *n* = 20), partial hepatectomy (*n* = 20), and pylorus-preserving pancreaticoduodenectomy (PPPD; *n* = 20). Activation of coagulation was assessed by levels of thrombin-antithrombin (TAT) complexes and D-dimer. Intrinsic activation was assessed with ELISA detecting free factor (F)XIIa and C1-esterase inhibitor bound to coagulation FXIIa, FXIa, and plasma kallikrein. Extrinsic activation was assessed by quantification of FVIIa-antithrombin complexes.

**Results:**

TAT and D-dimer were significantly elevated peri- and postoperatively in patients undergoing hepato-pancreato-biliary surgery. Markers of intrinsic pathway activation increased during OLT but not in patients undergoing partial hepatectomy or PPPD. Markers of extrinsic activation were low in all surgeries, even after adjustment for FVII zymogen levels. TAT and D-dimer were positively associated with intrinsic activation during OLT.

**Conclusion:**

This study provides evidence that enhanced activation of coagulation during liver transplantation is mediated by the intrinsic pathway of coagulation, whereas the route of coagulation activation in patients undergoing partial hepatectomy and PPPD remains unclear.

## Essentials

• Patients undergoing liver or pancreas surgeries face high clotting risks.
• This study investigated by which pathways clotting is activated in 3 major surgeries.
• Clotting is likely triggered via the intrinsic coagulation pathway in liver transplantation.
• New drugs against the intrinsic pathway may prevent clotting in liver transplantation.

## 1 Introduction

Patients undergoing hepato-pancreato-biliary (HPB) surgery may experience major alterations in their coagulation system [1,2]. Decreased plasma levels of pro- and anticoagulant proteins related to consumption or hemodilution have been described, along with increased levels of coagulation factor (F)VIII [[3], [4], [5]]. In addition, patients may already have preoperative hemostatic abnormalities related to the underlying liver disease or active cancer [3,6,7]. Routine laboratory tests of hemostasis, such as the prothrombin time, suggest that these patients develop a hypocoagulable state during and after the surgical procedure [3,[8], [9], [10]]. However, the prothrombin time is only sensitive to plasma levels of procoagulant proteins, and patients undergoing hepatobiliary surgery present with a concomitant decline in pro- and anticoagulant proteins [2,11]. More advanced hemostatic tests, such as thrombin generation tests and viscoelastic tests, for instance, thromboelastography or rotational thromboelastometry, demonstrate a normo- to hypercoagulable state in HPB patients [4,9,12]. Indeed, the risk for postoperative venous thromboembolism in HPB patients is high, even in the presence of adequate pharmacologic thromboprophylaxis [[13], [14], [15], [16]].

The mechanisms underlying the risk for venous thromboembolism in patients undergoing HPB surgery are underexplored. Perioperative activation of coagulation may occur because of surgical damage or local inflammatory responses. Such activation of coagulation may be an initial trigger for perioperative and early postoperative thrombotic events [17]. Understanding the mode of activation of coagulation may eventually allow more targeted thromboprophylaxis, which may reduce the incidence of venous thromboembolism. Coagulation during HPB surgery may be initiated via the extrinsic pathway due to exposure of extravascular tissue factor to the bloodstream following surgical injury [18]. Alternatively, or in addition, activation of the intrinsic pathway of coagulation may occur, for example, in response to thromboinflammatory processes in which agents such as polyphosphate or extracellular DNA initiate activation of the intrinsic pathway of coagulation [[19], [20], [21]].

To investigate the mode of activation of coagulation in patients before, during, and after HPB surgery, we examined markers of *in vivo* activation of coagulation and markers of intrinsic and extrinsic pathway activation.

## 2 Methods

### 2.1 Study design, setting, and participants

This observational cohort study retrospectively investigated temporal changes in hemostasis parameters in citrated plasma samples of patients who underwent HPB surgery, including orthotopic liver transplantation (OLT), partial hepatectomy, and pylorus-preserving pancreaticoduodenectomy (PPPD). To establish reference values, plasma samples of healthy controls were studied. Patients and controls were recruited from King’s College Hospital, London, between September 2017 and December 2017. Exclusion criteria were age < 18 years, acute liver failure, use of vitamin K antagonists, transfusion of blood products within the last 7 days, deep vein thrombosis within the last 30 days, hereditary thrombophilia or hemophilia, pregnancy, and positive HIV status. Participants provided written informed consent, and the study was approved by the National Research Ethics Service Committee London – Westminster (Study number 17/LO/0527) in accordance with the Declaration of Helsinki. This study followed the Strengthening the Reporting of Observational Studies in Epidemiology criteria [22].

### 2.2 Data collection

Details on patient characteristics, data collection, and blood sampling have been previously described by Bos et al. [23]. Briefly, blood samples were taken before anesthesia (baseline), at the end of surgery, and on postoperative days (PODs) 1, 3, and 6. Additional samples for OLT patients were taken 30 minutes after the start of the anhepatic phase and 30 minutes after reperfusion. Samples were taken into 3.2% sodium citrate tubes and centrifuged within 30 minutes after blood collection at 18 °C for 10 minutes at 2000 × *g* and then for 10 minutes at 10,000 × *g*. Then, samples were stored at −80 °C before further analysis.

### 2.3 Coagulation assays

Thrombin-antithrombin (TAT) levels were measured by a commercially available ELISA (Siemens), and D-dimer was measured on an automatic coagulation analyzer (StaCompact3) using reagents and protocols of the manufacturer (Stago). Intrinsic pathway activation was analyzed via nanobody-based ELISAs, as previously reported (Cosyne Kit, University Medical Center Utrecht) [6,24]. Specifically, C1-esterase inhibitor bound to activated FXII (C1inh-XIIa), activated FXI (C1inh-XIa), and plasma kallikrein (C1inh-PKa) were measured. Additionally, plasma levels of free (unbound) activated FXII (free XIIa) were examined. Extrinsic pathway activation was estimated by measuring levels of activated FVII bound to antithrombin (FVIIa-AT) using a commercially available ELISA (Asserachrom VIIa–AT, Stago). Levels of FVII and antithrombin were measured on the StaCompact3 using reagents and protocols of the manufacturer (Stago).

### 2.4 Bias

Efforts to address potential biases in this retrospective study included the use of clear inclusion and exclusion criteria to ensure a homogenous population, recruitment from a high-volume liver center for consistent management, and the establishment of reference values using healthy controls. While the sample size was limited by the availability of stored plasma, standardized protocols for sample collection, handling, and storage minimized variability. Confounding factors were mitigated by excluding patients with comorbidities affecting hemostasis, and all laboratory assays followed established methodologies. Detailed information on the race and ethnicity of participants was not available, which might limit the generalizability of our findings due to potential differences in coagulation dynamics based on sociocultural determinants.

### 2.5 Sample size

The study size was determined by the availability of existing citrated plasma samples and controls collected and stored in 2017 as part of earlier clinical research efforts. A formal power calculation could not be performed beforehand due to the absence of prior data on temporal changes in specific hemostasis parameters of the present study in this population.

### 2.6 Statistical analysis

Statistical analysis was performed using GraphPad Prism version 10.4.1. Variables were expressed as means ± SD, medians with IQR, or counts with percentages (%), as appropriate. Normality was assessed using the D'Agostino–Pearson test. To compare plasma levels of markers in patients against healthy controls or baseline levels, one-way analysis of variance (anova) with Šídák’s post hoc test or nonparametric one-way anova (Kruskal–Wallis test) with Dunn’s post hoc test was used, as appropriate. To determine correlations between patient plasma proteins, Pearson’s correlation coefficient with a simple linear regression was calculated. If the normality of one of the 2 variables was violated, Spearman’s correlation coefficient with a simple linear regression was calculated. A *P* value of ≤.05 was determined as statistically significant. Missing data were excluded from the analysis.

## 3 Results

### 3.1 Patient characteristics

We analyzed plasma samples from 60 HPB patients, of which 20 underwent OLT, 20 partial hepatectomy, and 20 PPPD. Patient data were compared against 41 healthy controls. Demographic, clinical, and laboratory characteristics of patients and controls have been described previously [23]. Of note, some data were missing, as blood could not be drawn from 2 OLT patients on POD3 and not from 8 partial hepatectomy patients and 7 PPPD patients on POD6 due to early discharge from the hospital.

### 3.2 Evidence of *in vivo* activation of coagulation in patients undergoing HPB surgery

**Figure 1:**
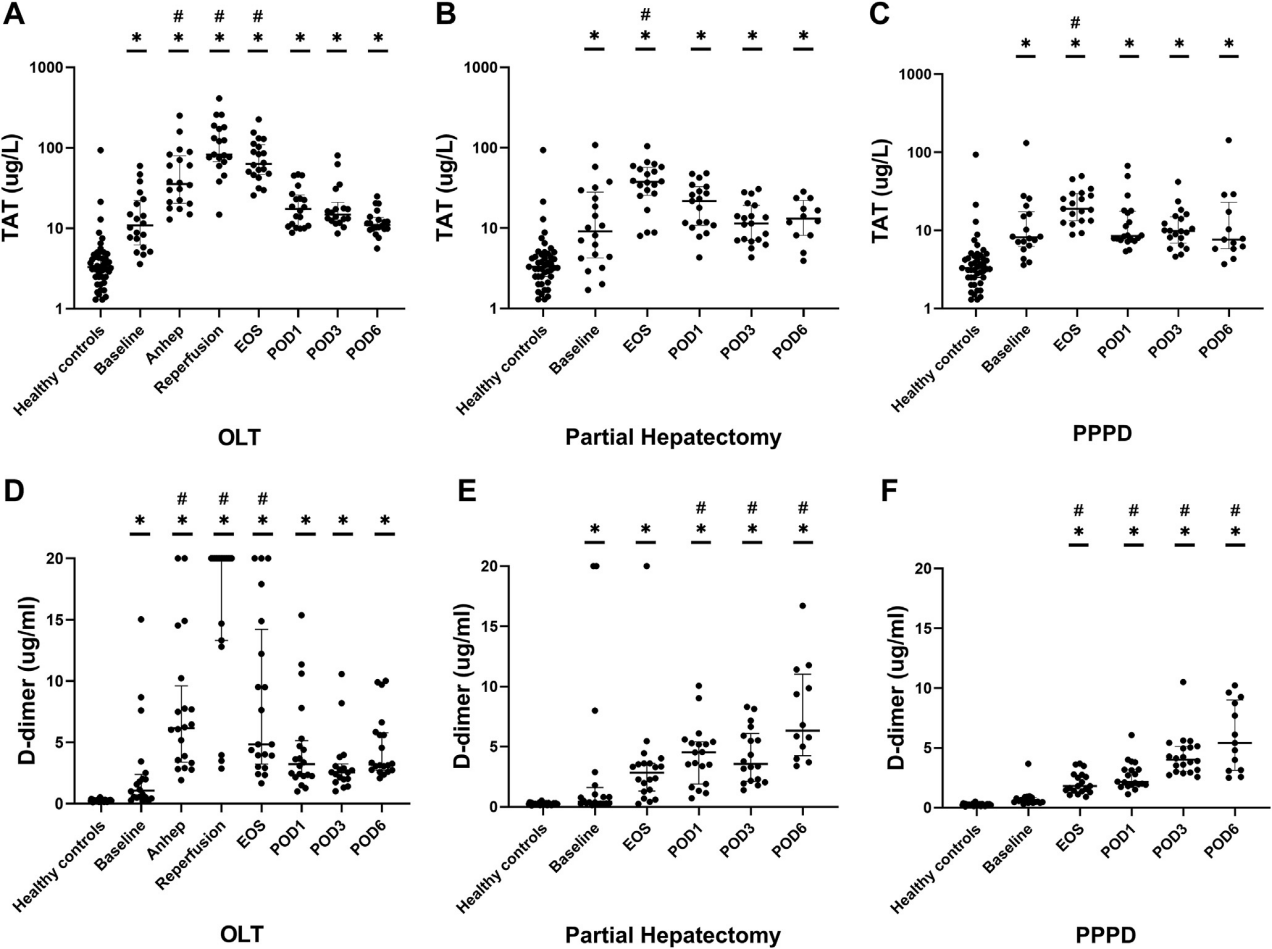
Activation of coagulation was estimated by plasma thrombin-antithrombin (TAT) levels (A–C) and D-dimer (D–F) in patients undergoing orthotopic liver transplantation (OLT; *n* = 20), partial hepatectomy (*n* = 20), and pylorus-preserving pancreaticoduodenectomy (PPPD; *n* = 20) compared with healthy controls (*n* = 41). Horizontal lines with error bars indicate medians with IQR. ∗*P* < .05 vs healthy controls, #*P* < .05 vs baseline. Anhep, 30 minutes after the start of the anhepatic phase; Baseline, after induction of anesthesia; EOS, at the end of surgery; Reperfusion, 30 minutes after reperfusion of the liver; POD, postoperative day.

To estimate the activation of coagulation in patients undergoing OLT, partial hepatectomy, or PPPD compared with healthy controls, we measured plasma levels of TAT and D-dimer. TAT levels were significantly higher at baseline in all 3 groups than in controls. TAT levels further increased toward the end of surgery and decreased thereafter. However, TAT levels remained significantly elevated until POD6, compared with controls (Figure 1A–C). In patients undergoing OLT (Figure 1D), D-dimer was slightly elevated at baseline, increased substantially during the anhepatic phase, and peaked after reperfusion. Thereafter, D-dimer decreased but was still higher than in controls at POD6. In patients undergoing partial hepatectomy (Figure 1E) but not PPPD (Figure 1F), D-dimer was elevated at baseline compared with controls. In both groups, D-dimer was increased at the end of surgery compared with healthy controls and progressively increased until POD6.

### 3.3 Activation of the intrinsic pathway during OLT, but not in partial hepatectomy and PPPD

**Figure 2:**
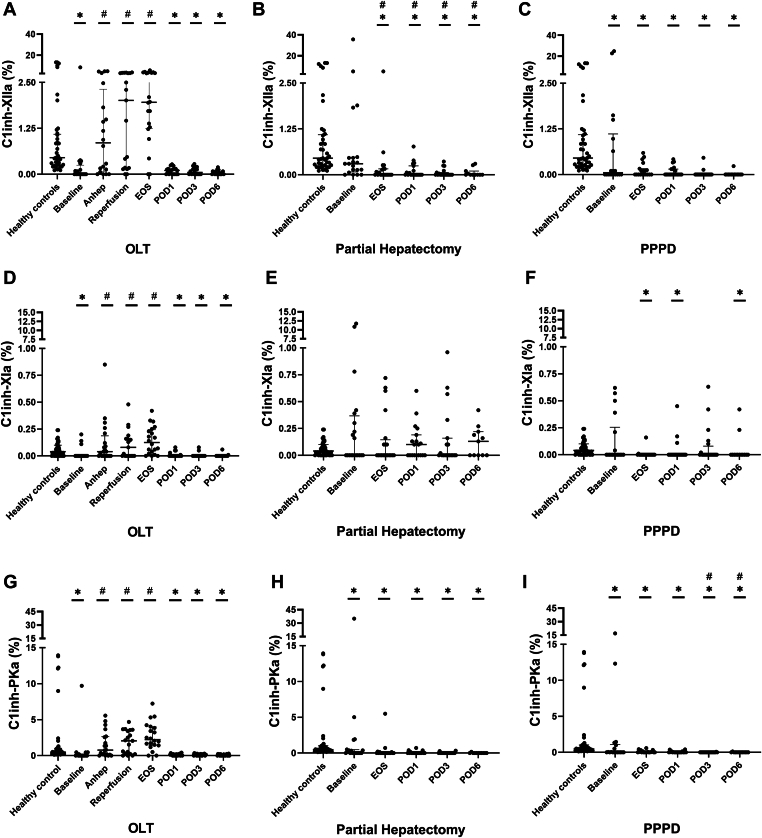
Intrinsic pathway activation was measured by C1 inhibitor bound to activated factor (F)XII (C1inh-XIIa; A–C), C1 inhibitor bound to activated FXI (C1inh-XIa; D–F), and C1 inhibitor bound to activated plasma kallikrein (C1inh-PKa; G–I) in patients undergoing orthotopic liver transplantation (OLT; *n* = 20), partial hepatectomy (*n* = 20), and pylorus-preserving pancreaticoduodenectomy (PPPD; *n* = 20) compared with healthy controls (*n* = 41). Horizontal lines with error bars indicate medians with IQR. ∗*P* < .05 vs healthy controls, #*P* < .05 vs baseline. Anhep, 30 minutes after the start of the anhepatic phase; Baseline, after induction of anesthesia; EOS, at the end of surgery; Reperfusion, 30 minutes after reperfusion of the liver; POD, postoperative day.

**Figure 3:**
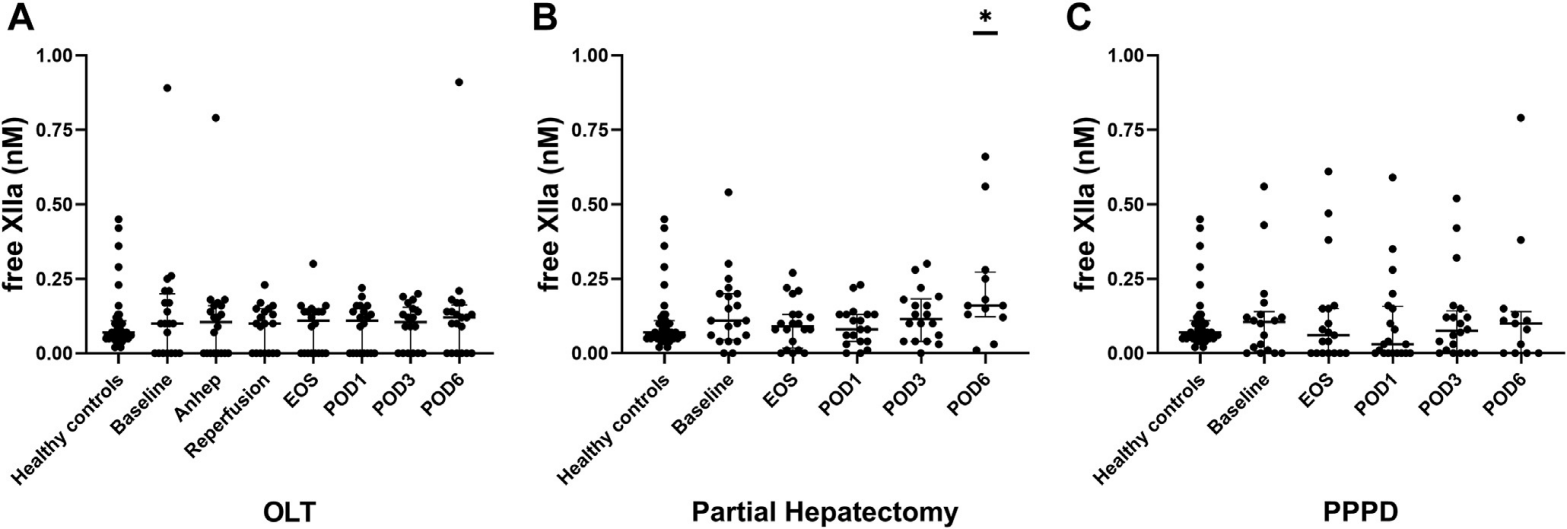
Intrinsic pathway activation was also estimated by plasma levels of free (unbound) activated factor XII (free XIIa) in patients undergoing orthotopic liver transplantation (OLT; A, *n* = 20), partial hepatectomy (B, *n* = 20), and pylorus-preserving pancreaticoduodenectomy (PPPD; C, *n* = 20) compared with healthy controls (*n* = 41). Horizontal lines with error bars indicate medians with IQR. ∗*P* < .05 vs healthy controls, #*P* < .05 vs baseline. Anhep, 30 minutes after the start of the anhepatic phase; Baseline, after induction of anesthesia; EOS, at the end of surgery; Reperfusion, 30 minutes after reperfusion of the liver; POD, postoperative day.

In OLT patients, C1inh-XIIa, C1inh-XIa, and C1inh-PKa (Figure 2A, D, G) were low at baseline, increased perioperatively, peaked at reperfusion and the end of surgery, and were low again at POD1 to 6. However, the perioperative elevation was only significant compared with baseline levels and not with controls. In patients undergoing partial hepatectomy (Figure 2B, E, H) and PPPD (Figure 2C, F, I), peri- and postoperative levels of C1inh-XII, C1inh-XI, and C1inh-PKa were similar to or slightly lower than in controls. Besides a significant increase on POD6 after partial hepatectomy compared with controls, levels of free XIIa (Figure 3A–C) did not significantly change in patients undergoing HPB surgery.

### 3.4 No evidence of extrinsic pathway activation during HPB surgery

**Figure 4:**
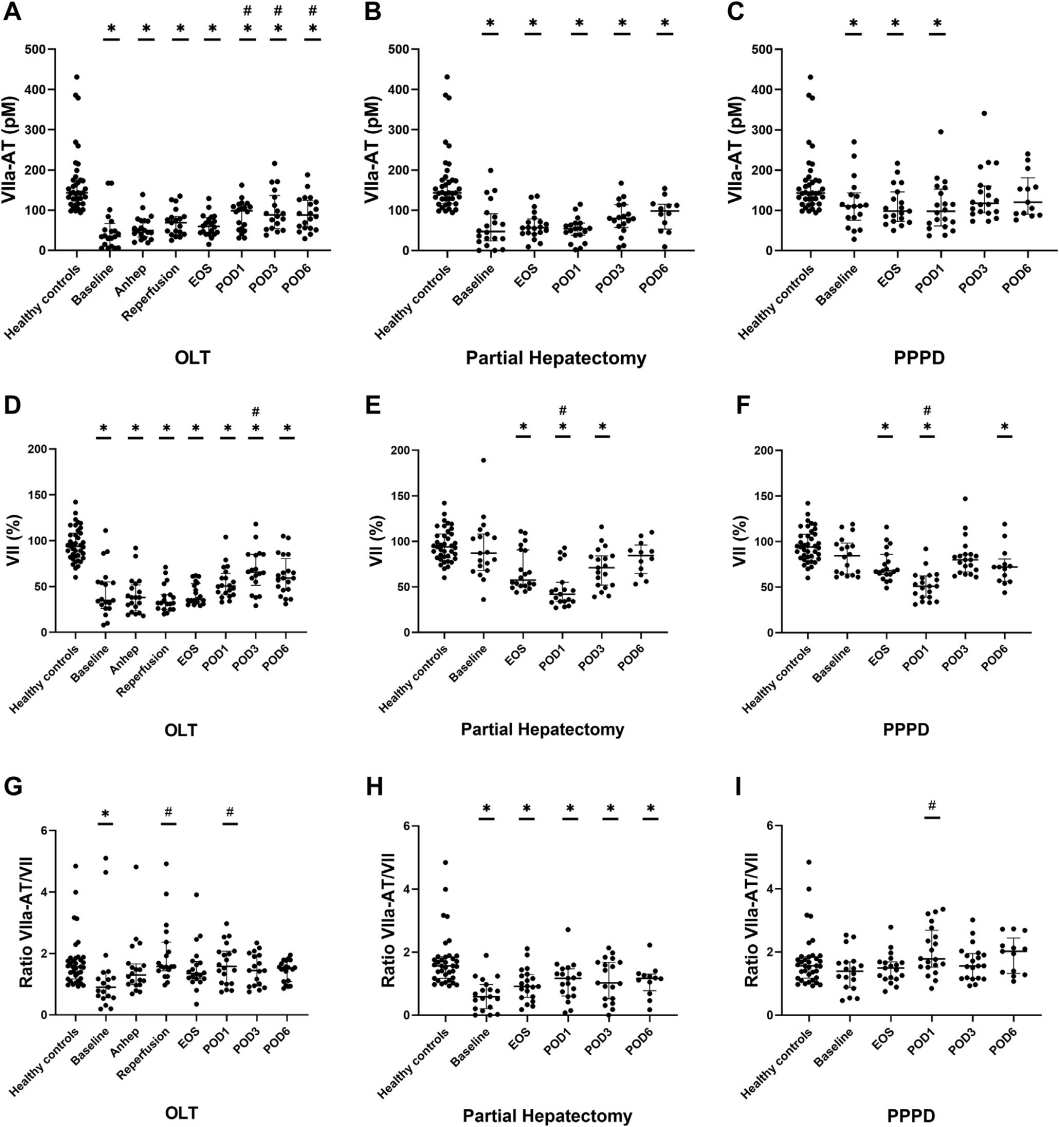
Extrinsic pathway activation was measured by plasma levels of activated factor (F)VII bound to antithrombin (FVIIa-AT; A–C), plasma levels of FVII (VII; D–F), and a calculated ratio of FVIIa-AT corrected for FVII levels (FVIIa-AT/VII; G–I) in patients undergoing orthotopic liver transplantation (OLT; *n* = 20), partial hepatectomy (*n* = 20), and pylorus-preserving pancreaticoduodenectomy (PPPD; *n* = 20) compared with healthy controls (*n* = 41). Horizontal lines with error bars indicate medians with IQR. ∗*P* < .05 vs healthy controls, #*P* < .05 vs baseline. Anhep, 30 minutes after the start of the anhepatic phase; Baseline, after induction of anesthesia; EOS, at the end of surgery; Reperfusion, 30 minutes after reperfusion of the liver; POD, postoperative day.

To estimate extrinsic pathway activation, we measured plasma levels of FVIIa-AT and FVII. Baseline levels of FVIIa-AT (Figure 4A–C) were low in HPB patients and increased slightly until POD6 but remained significantly lower than in healthy controls at all time points. Baseline levels of FVII (Figure 4D–F) were reduced in HPB patients compared with controls and remained lower than in controls up until POD6. As FVII levels were lower during all types of surgery than those of controls, we analyzed the ratio of FVIIa-AT and FVII levels. The ratio of FVIIa-AT/FVII (Figure 4G–I) in HPB patients was similar to or lower than the ratio in healthy controls.

### 3.5 Deficiency of antithrombin during and after HPB surgery

**Figure 5:**
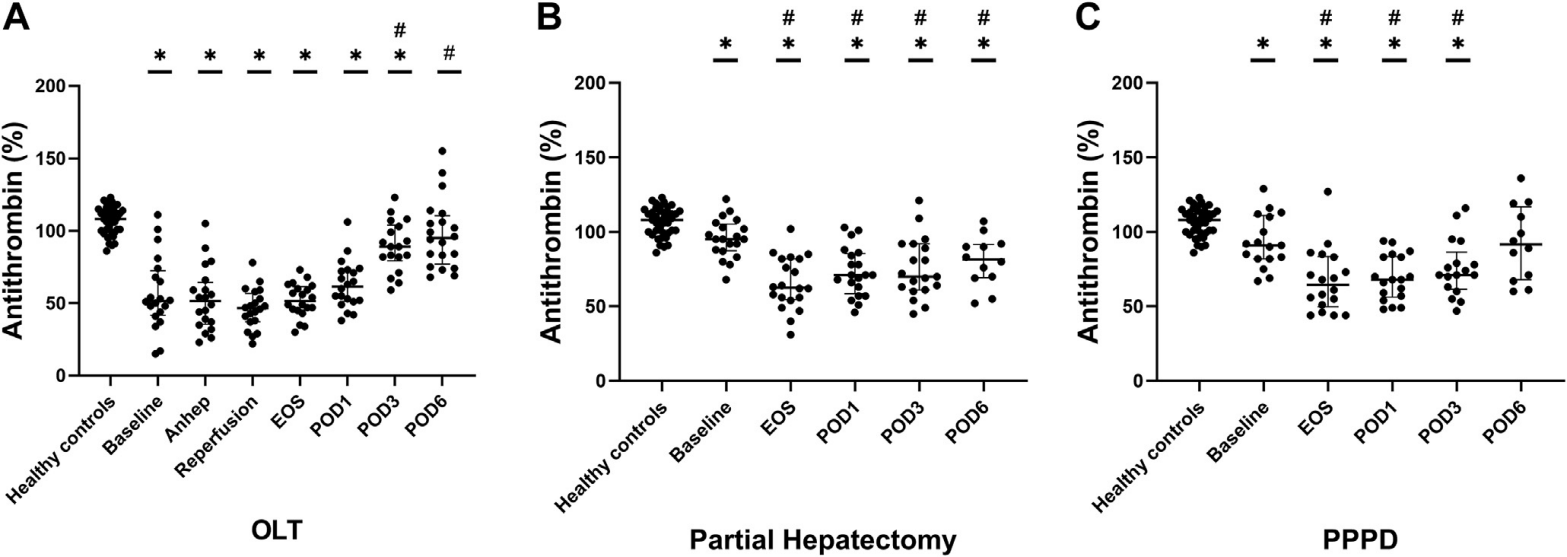
Natural anticoagulant levels were estimated by plasma levels of antithrombin in patients undergoing orthotopic liver transplantation (OLT; A, *n* = 20), partial hepatectomy (B, *n* = 20), and pylorus-preserving pancreaticoduodenectomy (PPPD; C, *n* = 20) compared with healthy controls (*n* = 41). Horizontal lines with error bars indicate medians with IQR. ∗*P* < .05 vs healthy controls, #*P* < .05 vs baseline. Anhep, 30 minutes after the start of the anhepatic phase; Baseline, after induction of anesthesia; EOS, at the end of surgery; Reperfusion, 30 minutes after reperfusion of the liver; POD, postoperative day.

Antithrombin levels were substantially decreased at baseline in patients undergoing OLT (Figure 5A) and progressively increased from the end of surgery onward, but always remained below the levels of controls. In patients undergoing partial hepatectomy (Figure 5B) and PPPD (Figure 5C), antithrombin levels were slightly lower than those in controls at the start of surgery, decreased perioperatively, and slightly increased on POD1 to 6, but remained lower than in controls.

### 3.6 Correlations between markers of *in vivo* activation of coagulation and markers of intrinsic and extrinsic pathway activation

In intraoperative samples of patients undergoing OLT, we found significant but weak positive correlations between levels of TAT and D-dimer and markers of intrinsic activation, except for free XIIa (Supplementary Figure 1A–D and 2A–D). Intraoperative levels of TAT and D-dimer did not significantly correlate with FVIIa-AT complex levels, but FVIIa-AT/FVII levels had a statistically significant but weak positive correlation with D-dimer (Supplementary Figure 1E–H and 2E–H). In postoperative samples, there were no significant correlations between markers of activation of coagulation and markers of extrinsic or intrinsic activation in patients undergoing OLT (Supplementary Figures 3 and 4). Furthermore, in patients undergoing partial hepatectomy, end-of-surgery levels of FVIIa-AT and FVIIa-AT/FVII moderately positively correlated with levels of TAT (Supplementary Figure 5). Although intraoperative TAT levels did not show significant correlations with markers of intrinsic activation in partial hepatectomy, there was a significant, moderately positive correlation between intraoperative D-dimer and C1inh-XIa (Supplementary Figure 6). Postoperatively in patients undergoing partial hepatectomy, only C1inh-PKa showed a significantly weak positive correlation with D-dimer (Supplementary Figures 7 and 8). In patients undergoing PPPD, intraoperative levels of FVIIa-AT, but not FVIIa-AT/FVII, correlated moderately positively with levels of TAT (Supplementary Figure 9). However, no correlations between intraoperative D-dimer and markers of intrinsic or extrinsic activation were observed (Supplementary Figure 10). In postoperative samples of PPPD patients, levels of free XIIa correlated weakly positively and significantly with D-dimer (Supplementary Figures 11 and 12).

## 4 Discussion

In this study, we found substantial *in vivo* activation of coagulation with concurrent intrinsic pathway activation in patients during OLT, as evidenced by elevated levels of TAT, D-dimer, and markers of intrinsic pathway activation. Additionally, markers of activation of coagulation positively correlated with markers of intrinsic pathway activation during OLT. Activation of coagulation also occurred in patients undergoing partial hepatectomy and PPPD, but without detectable intrinsic or extrinsic pathway activation. Our findings are in line with several studies showing activation of coagulation during HPB surgery [5,[25], [26], [27]] while providing a novel mechanistic insight, ie, the intrinsic pathway is a driver of coagulation activation in patients undergoing OLT.

Activation of coagulation was most profound in patients undergoing OLT and peaked after the anhepatic and reperfusion phases, which is consistent with prior studies on peri- and early postoperative increases in TAT and D-dimer [25,26,[28], [29], [30], [31], [32]]. We confirm and extend previous findings on the activation of coagulation in patients undergoing partial hepatectomy, and we are, to the best of our knowledge, the first to formally document the *in vivo* activation of coagulation in PPPD patients [5,27,[33], [34], [35]]. Several prothrombotic mechanisms might have initiated or propagated the activation of coagulation during HPB surgery. First, surgical damage and related inflammatory responses are likely responsible for the activation of coagulation. Second, in OLT patients, reduced hepatic clearance could have caused the accumulation of activated coagulation proteins during the anhepatic phase, which exacerbates the activation of coagulation. In addition, endothelial activation caused by general surgical stress and reperfusion injury may drive coagulation activation either directly by prothrombotic properties of the activated endothelium or indirectly via endothelial release of hemostatic proteins [28,32,36]. Third, a deficiency in natural anticoagulants may have amplified initial coagulation activation. Indeed, we found decreased antithrombin levels until POD6, confirming previous studies on reductions in natural anticoagulants up to POD14 [6,26,33,37]. Fourth, peri- and postoperative activation of coagulation in our HPB patients might have been exacerbated by ongoing preoperative activation of coagulation, likely due to the underlying end-stage liver disease or liver cancer [6,27,38,39], as indicated by increased TAT and D-dimer at baseline. Unlike TAT levels, D-dimer progressively increased postoperatively in partial hepatectomy and PPPD, confirming previous studies [35,[40], [41], [42], [43]]. As the half-life of D-dimer is approximately 8 hours [44], this finding indicates additional major clot formation in the postoperative period, which has been previously demonstrated [45]. Overall, our findings suggest ongoing activation of coagulation during and after OLT, partial hepatectomy, and PPPD, which may translate into an increased risk for venous thromboembolism throughout the surgical and postoperative periods.

Intrinsic pathway activation during OLT likely initiated activation of coagulation, as indicated by a positive correlation of TAT and D-dimer with elevated levels of C1inh-XIIa, C1inh-XIa, and C1inh-PKa. Of note, levels of free XIIa in OLT were low in contrast to C1inh-XIIa, which may be explained by the rapid inactivation of free XIIa by its natural inhibitors in plasma [46]. Although previous studies have provided evidence of activation of the intrinsic pathway during human liver transplantation [47,48], this is the first study using an extensive panel of markers of *in vivo* activation of this contact pathway. We propose 3 mechanisms that might have led to increased intrinsic activation. First, contact of blood with foreign surfaces results in the activation of the intrinsic pathway [49]. Such foreign surfaces include surfaces of surgical instruments, vascular clamps, and cannulas that are used during liver transplantation [19,49,50]. Second, surgical trauma might have led to the disruption of endothelial cells, particularly after the reperfusion phase [25], exposing subendothelial collagen and extracellular DNA, which can activate intrinsic factors [51]. Third, surgical trauma may also induce the release of polyphosphate from activated platelets, contributing to intrinsic pathway activation [20,52,53]. Although surgical trauma occurs during partial hepatectomy and PPPD and despite moderate activation of coagulation, we found no evidence of intrinsic pathway activation in these patients. We speculate that, as these surgeries are less invasive than OLT, contact activation and surgical trauma are not sufficient to cause intrinsic activation. Alternatively, we might have missed the peak of intrinsic activation during the procedures, as samples were only taken at the beginning and end of surgery.

Despite moderate activation of coagulation, no detectable extrinsic activation occurred in HPB patients, which is surprising as surgical injury is known to induce exposure of extravascular tissue factor to the bloodstream in major abdominal surgery [18]. Local extrinsic activation may not lead to detectable elevations in FVIIa-AT complexes in peripheral blood, and future studies may examine markers of intrinsic and extrinsic activation in samples taken directly from the surgical field. We speculate that low-grade extrinsic activation could have occurred in all HPB patients. This low-grade activation may have contributed to thrombin generation, as a small initiating signal may have been amplified due to deficiencies in natural anticoagulants. Such a mechanism was previously suggested to drive *in vivo* thrombin generation in patients with end-stage liver disease by our group [6]. Furthermore, the comparison of markers of activation of coagulation in patients undergoing partial hepatectomy or PPPD without underlying liver disease with patients undergoing liver transplantation may be complicated, as these markers are cleared by the liver. Prior to and during liver transplantation and following partial liver resection, clearance of these markers may be impaired, leading to the (temporal) accumulation of these markers.

Our data thus suggest that thrombin generation in patients undergoing OLT is primarily driven by intrinsic activation of coagulation, whereas the mechanism driving thrombin generation during partial hepatectomy and PPPD requires further study. Although it needs to be studied whether coagulation activation via intrinsic activation increases the risk for perioperative thrombotic complications, our findings may have direct clinical relevance. Given the high risk of venous thromboembolism in HPB surgery, even in the presence of optimal thromboprophylaxis, alternative antithrombotic strategies are of definite interest. Inhibitors of the intrinsic pathway, such as agents targeting FXI or FXII that are in current clinical development [54], may be particularly useful in reducing the risk of venous thromboembolism in patients undergoing HPB surgery.

## 5 Conclusions

In conclusion, *in vivo* activation of coagulation is enhanced in patients during and after OLT and likely proceeds through increased activation of the intrinsic pathway, possibly resulting in an increased risk for perioperative thrombotic complications. Activation of coagulation in patients undergoing partial hepatectomy and PPPD is also elevated, but the underlying route of activation remains unclear.
