# Supplementary material for: In vivo activation of coagulation during human liver transplantation is associated with activation of the intrinsic pathway: an observational cohort study
Source: Res Pract Thromb Haemost. 2025 Apr 24;9(4):102872. doi: 10.1016/j.rpth.2025.102872 (PMC12149591; doi:10.1016/j.rpth.2025.102872)
Supplement: Supplementary Material [file mmc1.docx]

**In vivo activation of coagulation during human liver transplantation is associated with activation of the intrinsic pathway: An observational cohort study**

Fynn L. Elvers^1^, Jelle Adelmeijer^1^, Sarah Bos^2^, Coen Maas^3^, William Bernal^4^, Ton Lisman^1^

*^1^ Surgical Research Laboratory and Section of Hepatobiliary Surgery and Liver Transplantation, Department of Surgery, University of Groningen, University Medical Center Groningen, Groningen, The Netherlands; ^2^ Department of Gastroenterology, Treant Hospital, Emmen, The Netherlands;*

*^3^ Central Diagnostic Laboratory Research, University Medical Center Utrecht, Utrecht University, Utrecht, The Netherlands; ^4^ Liver Intensive Therapy Unit, Institute of Liver Studies, Kings College Hospital, Denmark Hill, London SE5 9RS, United Kingdom.*

**Contact information**

Ton Lisman

University Medical Center Groningen

Department of Surgery, Surgical Research Laboratory and Section of Hepatobiliary Surgery and Liver Transplantation

BA33, Hanzeplein 1, 9713 GZ Groningen, The Netherlands

Tel: +31-50-361-9028, Fax: +31-50-363-2796

Email: [j.a.lisman@umcg.nl](mailto:j.a.lisman@umcg.nl)

**Supplementary figure 1.** Correlations between intra-operative levels of TAT and C1inh-XIIa (A), C1inh-XIa (B), C1inh-PKa (C), free XIIa (D), VIIa-AT (E), VII (F), VIIa-AT/VII (G), and AT (H) in patients undergoing orthotopic liver transplantation. Intra-operative datapoints of serial plasma samples include anhepatic phase, reperfusion, and end of surgery. Pearson or Spearman correlation coefficients with corresponding p-values are displayed. Abbreviations: TAT, Thrombin-Antithrombin complexes; C1inh-XIIa, C1 inhibitor bound to activated Factor XII; C1inh-XIa, C1 inhibitor bound to activated Factor XI; C1inh-PKa, C1 inhibitor bound to activated Plasma kallikrein; VIIa-AT, Activated Factor VII bound to Antithrombin; VII, Factor VII; AT, Antithrombin.

**Supplementary figure 2.** Correlations between intra-operative levels of D-dimers and C1inh-XIIa (A), C1inh-XIa (B), C1inh-PKa (C), free XIIa (D), VIIa-AT (E), VII (F), VIIa-AT/VII (G), and AT (H) in patients undergoing orthotopic liver transplantation. Intra-operative datapoints of serial plasma samples include anhepatic phase, reperfusion, and end of surgery. Pearson or Spearman correlation coefficients with corresponding p-values are displayed. Abbreviations: TAT, Thrombin-Antithrombin complexes; C1inh-XIIa, C1 inhibitor bound to activated Factor XII; C1inh-XIa, C1 inhibitor bound to activated Factor XI; C1inh-PKa, C1 inhibitor bound to activated Plasma kallikrein; VIIa-AT, Activated Factor VII bound to Antithrombin; VII, Factor VII; AT, Antithrombin.

**Supplementary figure 3.** Correlations between post-operative levels of TAT and C1inh-XIIa (A), C1inh-XIa (B), C1inh-PKa (C), free XIIa (D), VIIa-AT (E), VII (F), VIIa-AT/VII (G), and AT (H) in patients undergoing orthotopic liver transplantation. Post-operative datapoints of serial plasma samples include post-operative days 1, 3 and 6. Pearson or Spearman correlation coefficients with corresponding p-values are displayed. Abbreviations: C1inh-XIIa, C1 inhibitor bound to activated Factor XII; C1inh-XIa, C1 inhibitor bound to activated Factor XI; C1inh-PKa, C1 inhibitor bound to activated Plasma kallikrein; VIIa-AT, Activated Factor VII bound to Antithrombin; VII, Factor VII; AT, Antithrombin.

**Supplementary figure 4.** Correlations between post-operative levels of D-dimers and C1inh-XIIa (A), C1inh-XIa (B), C1inh-PKa (C), free XIIa (D), VIIa-AT (E), VII (F), VIIa-AT/VII (G), and AT (H) in patients undergoing orthotopic liver transplantation. Post-operative datapoints of serial plasma samples include post-operative days 1, 3 and 6. Pearson or Spearman correlation coefficients with corresponding p-values are displayed. Abbreviations: C1inh-XIIa, C1 inhibitor bound to activated Factor XII; C1inh-XIa, C1 inhibitor bound to activated Factor XI; C1inh-PKa, C1 inhibitor bound to activated Plasma kallikrein; VIIa-AT, Activated Factor VII bound to Antithrombin; VII, Factor VII; AT, Antithrombin.

**Supplementary figure 5.** Correlations between intra-operative levels of TAT and C1inh-XIIa (A), C1inh-XIa (B), C1inh-PKa (C), free XIIa (D), VIIa-AT (E), VII (F), VIIa-AT/VII (G), and AT (H) in patients undergoing hemi-hepatectomy. Intra-operative datapoints of serial plasma samples include end of surgery. Pearson or Spearman correlation coefficients with corresponding p-values are displayed. Abbreviations: TAT, Thrombin-Antithrombin complexes; C1inh-XIIa, C1 inhibitor bound to activated Factor XII; C1inh-XIa, C1 inhibitor bound to activated Factor XI; C1inh-PKa, C1 inhibitor bound to activated Plasma kallikrein; VIIa-AT, Activated Factor VII bound to Antithrombin; VII, Factor VII; AT, Antithrombin.

**Supplementary figure 6.** Correlations between intra-operative levels of D-dimers and C1inh-XIIa (A), C1inh-XIa (B), C1inh-PKa (C), free XIIa (D), VIIa-AT (E), VII (F), VIIa-AT/VII (G), and AT (H) in patients undergoing hemi-hepatectomy. Intra-operative datapoints of serial plasma samples include end of surgery. Pearson or Spearman correlation coefficients with corresponding p-values are displayed. Abbreviations: TAT, Thrombin-Antithrombin complexes; C1inh-XIIa, C1 inhibitor bound to activated Factor XII; C1inh-XIa, C1 inhibitor bound to activated Factor XI; C1inh-PKa, C1 inhibitor bound to activated Plasma kallikrein; VIIa-AT, Activated Factor VII bound to Antithrombin; VII, Factor VII; AT, Antithrombin.

**Supplementary figure 7.** Correlations between post-operative levels of TAT and C1inh-XIIa (A), C1inh-XIa (B), C1inh-PKa (C), free XIIa (D), VIIa-AT (E), VII (F), VIIa-AT/VII (G), and AT (H) in patients undergoing hemi-hepatectomy. Post-operative datapoints of serial plasma samples include post-operative days 1, 3 and 6. Pearson or Spearman correlation coefficients with corresponding p-values are displayed. Abbreviations: TAT, Thrombin-Antithrombin complexes; C1inh-XIIa, C1 inhibitor bound to activated Factor XII; C1inh-XIa, C1 inhibitor bound to activated Factor XI; C1inh-PKa, C1 inhibitor bound to activated Plasma kallikrein; VIIa-AT, Activated Factor VII bound to Antithrombin; VII, Factor VII; AT, Antithrombin.

**Supplementary figure 8.** Correlations between post-operative levels of D-dimers and C1inh-XIIa (A), C1inh-XIa (B), C1inh-PKa (C), free XIIa (D), VIIa-AT (E), VII (F), VIIa-AT/VII (G), and AT (H) in patients undergoing hemi-hepatectomy. Post-operative datapoints of serial plasma samples include post-operative days 1, 3 and 6. Pearson or Spearman correlation coefficients with corresponding p-values are displayed. Abbreviations: C1inh-XIIa, C1 inhibitor bound to activated Factor XII; C1inh-XIa, C1 inhibitor bound to activated Factor XI; C1inh-PKa, C1 inhibitor bound to activated Plasma kallikrein; VIIa-AT, Activated Factor VII bound to Antithrombin; VII, Factor VII; AT, Antithrombin.

**Supplementary figure 9.** Correlations between intra-operative levels of TAT and C1inh-XIIa (A), C1inh-XIa (B), C1inh-PKa (C), free XIIa (D), VIIa-AT (E), VII (F), VIIa-AT/VII (G), and AT (H) in patients undergoing pylorus-preserving pancreatoduodenectomy. Intra-operative datapoints of serial plasma samples include end of surgery. Pearson or Spearman correlation coefficients with corresponding p-values are displayed. Abbreviations: TAT, Thrombin-Antithrombin complexes; C1inh-XIIa, C1 inhibitor bound to activated Factor XII; C1inh-XIa, C1 inhibitor bound to activated Factor XI; C1inh-PKa, C1 inhibitor bound to activated Plasma kallikrein; VIIa-AT, Activated Factor VII bound to Antithrombin; VII, Factor VII; AT, Antithrombin.

**Supplementary figure 10.** Correlations between intra-operative levels of D-dimers and C1inh-XIIa (A), C1inh-XIa (B), C1inh-PKa (C), free XIIa (D), VIIa-AT (E), VII (F), VIIa-AT/VII (G), and AT (H) in patients undergoing pylorus-preserving pancreatoduodenectomy. Intra-operative datapoints of serial plasma samples include end of surgery. Pearson or Spearman correlation coefficients with corresponding p-values are displayed. Abbreviations: C1inh-XIIa, C1 inhibitor bound to activated Factor XII; C1inh-XIa, C1 inhibitor bound to activated Factor XI; C1inh-PKa, C1 inhibitor bound to activated Plasma kallikrein; VIIa-AT, Activated Factor VII bound to Antithrombin; VII, Factor VII; AT, Antithrombin.

**Supplementary figure 11.** Correlations between post-operative levels of TAT and C1inh-XIIa (A), C1inh-XIa (B), C1inh-PKa (C), free XIIa (D), VIIa-AT (E), VII (F), VIIa-AT/VII (G), and AT (H) in patients undergoing pylorus-preserving pancreatoduodenectomy. Post-operative datapoints of serial plasma samples include post-operative days 1, 3, and 6. Pearson or Spearman correlation coefficients with corresponding p-values are displayed. Abbreviations: TAT, Thrombin-Antithrombin complexes; C1inh-XIIa, C1 inhibitor bound to activated Factor XII; C1inh-XIa, C1 inhibitor bound to activated Factor XI; C1inh-PKa, C1 inhibitor bound to activated Plasma kallikrein; VIIa-AT, Activated Factor VII bound to Antithrombin; VII, Factor VII; AT, Antithrombin.

**Supplementary figure 12.** Correlations between post-operative levels of D-dimers and C1inh-XIIa (A), C1inh-XIa (B), C1inh-PKa (C), free XIIa (D), VIIa-AT (E), VII (F), VIIa-AT/VII (G), and AT (H) in patients undergoing pylorus-preserving pancreatoduodenectomy. Post-operative datapoints of serial plasma samples include post-operative days 1, 3, and 6. Pearson or Spearman correlation coefficients with corresponding p-values are displayed. Abbreviations: C1inh-XIIa, C1 inhibitor bound to activated Factor XII; C1inh-XIa, C1 inhibitor bound to activated Factor XI; C1inh-PKa, C1 inhibitor bound to activated Plasma kallikrein; VIIa-AT, Activated Factor VII bound to Antithrombin; VII, Factor VII; AT, Antithrombin.
